# Risk Factors and Outcome of Multidrug-Resistant Infections after Heart Transplant: A Contemporary Single Center Experience

**DOI:** 10.3390/microorganisms9061210

**Published:** 2021-06-03

**Authors:** Arta Karruli, Jacopo de Cristofaro, Roberto Andini, Domenico Iossa, Mariano Bernardo, Cristiano Amarelli, Irene Mattucci, Rosa Zampino, Raffaele Zarrilli, Emanuele Durante-Mangoni

**Affiliations:** 1 Department of Precision Medicine, University of Campania ‘L. Vanvitelli’, 80138 Naples, Italy; arta.karruli@unicampania.it (A.K.); jacopo@decristofaro.info (J.d.C.); domenico.iossa@unicampania.it (D.I.); 2 Unit of Infectious and Transplant Medicine, AORN Ospedali dei Colli-Monaldi Hospital, 80131 Naples, Italy; roberto.andini@ospedalideicolli.it (R.A.); rosa.zampino@unicampania.it (R.Z.); 3 Unit of Microbiology & Virology, AORN Ospedali dei Colli, 80131 Naples, Italy; mariano.bernardo@ospedalideicolli.it; 4 Unit of Cardiac Transplant, AORN Ospedali dei Colli-Monaldi Hospital, 80131 Naples, Italy; cristiano.amarelli@ospedalideicolli.it (C.A.); irene.mattucci@ospedalideicolli.it (I.M.); 5 Department of Advanced Medical and Surgical Sciences, University of Campania ‘L. Vanvitelli’, 80138 Naples, Italy; 6 Department of Public Health, University of Naples Federico II, 80138 Naples, Italy; rafzarri@unina.it

**Keywords:** MDR, XDR, infection, heart transplant, risk factors, hospitalization, outcome

## Abstract

(1) Background: The aim of this study was to assess risk factors for multidrug-resistant/extensively drug-resistant (MDR/XDR) bacterial infections in heart transplant (HT) patients within three months after surgery and its impact on patient outcome. (2) Methods: Retrospective analysis of clinical, hemato-chemical, imaging, treatment and outcome data from 47 heart transplant recipients from January 2016 to December 2018. MDR/XDR infections were compared to non-MDR/XDR and noninfected patients. (3) Results: Most participants were males, median age 51 years: 35 (74.5%) developed an infection after HT; 14 (29.8%) were MDR/XDR infections. Prolonged hospital stay before HT correlated to MDR/XDR infection (*p* < 0.001). Sequential organ failure assessment (SOFA) score at sampling day was higher in MDR/XDR (*p* = 0.027). MDR/XDR were mostly blood-stream (BSI) (*p* = 0.043) and skin-soft tissue (SSTI) (*p* = 0.047) infections. Gram-negative infections were the most frequent, specifically carbapenem-resistant *Klebsiella pneumoniae*. Antibiotic therapy duration for MDR/XDR infections was longer (*p* = 0.057), eradication rate lower (*p* = 0.083) and hospital stay longer (*p* = 0.005) but not associated with a worse outcome. (4) Conclusions: MDR/XDR infections affect compromised HT recipients with a history of prolonged hospitalization, causing a lower rate of eradication and increased hospital stay. These frequently present as BSI and SSTI. We emphasize the need to prevent contamination of central venous catheters and the surgical site.

## 1. Introduction

Heart transplantation is currently considered the treatment of choice for end-stage heart failure, showing the best short- and long-term clinical outcomes [1,2]. Most transplant candidates present compromised health conditions due to primary organ disease as well as various comorbidities [3]. Immediately following transplant, a pharmacologically-induced immune suppressive state ensues and, while under-immune suppression, may result in organ rejection [4]. Over-immune suppression may pose patients at an increased risk of infection, still a major cause of morbidity and mortality after surgery [5]. In fact, hospital mortality of heart transplant recipients may be as high as 10%, with organ rejection and infections remaining the major causes of an unfavorable outcome [3,6].

The most common infections following heart transplant are bacterial in origin, followed by viral infections [6,7]. Multidrug-resistant (MDR) infections may occur in up to 20% of heart transplant recipients during the initial 6 months post-transplant [7]. The most common MDR pathogens affecting heart transplant recipients within a year after surgery are extended-spectrum beta-lactamase (ESBL) producing *Klebsiella pneumonia* and methicillin-resistant *Staphylococcus aureus* (MRSA) [8]. Apart from immune suppression, other factors that influence MDR infection development in non-transplant patients also play a role in transplant recipients, including surgery [9] and intensive care unit (ICU) stay [10]. In fact, MDR infection rates in ICU non transplanted patients range from 14% to nearly 50% [11,12,13].

The epidemiology and risk factors for the development of MDR infections have been recently assessed in abdominal organ transplant recipients and include prolonged hospital stay and extended prior antibiotic treatment [14,15]. In contrast, limited data are currently available on the drivers and prognosis of MDR infection in heart transplant recipients.

Therefore, we carried out this study with the aim of assessing the risk factor for developing MDR infections following heart transplant and evaluating their effect on recipient outcome.

## 2. Patients and Methods

### 2.1. Study Design

This was a retrospective, observational study. All patients who underwent orthotopic heart transplantation at the Transplant Center of the V. Monaldi Hospital in Naples, Italy, between January 2016 and December 2018 were included in this study. Data regarding the clinical characteristics of patients, immunosuppressive regimens, the onset of graft rejection and infections, as well as clinical features, microbiological diagnosis and outcomes of infectious episodes, were recorded. Infections with a microbiological diagnosis and episodes without a microbiological diagnosis, but with definite clinical signs of infection coupled with biochemical inflammatory parameter elevation, and occurred within the first 3 months after transplantation, were considered in this analysis. Surveillance microbiological sampling of blood, urines, airways and surgical wounds was performed in all patients at least once weekly and additional cultures were performed as dictated by patient conditions. This study was approved by the Ethics Committee of the University of Campania ‘Luigi Vanvitelli’ and the AORN Ospedali dei Colli on April 18, 2018 with protocol n. 307/2018.

### 2.2. Definitions

Antimicrobial susceptibilities of microbial isolates were performed using the Vitek 2 system and the AST-GN card (bioMérieux, Marcy l’Etoile, France). Values were interpreted according to a breakpoint table for the interpretation of MIC values and zone diameters (European Committee on Antimicrobial Susceptibility Testing, 2015) [16].

Infections were classified as being due to “multidrug-resistant” (MDR), “extensively drug-resistant” (XDR) or “pan-drug-resistant” (PDR) pathogens, and compared with those caused by drug-sensitive/non-MDR pathogens, in accordance with the definitions of Magiorakos et al. [17]. Accordingly, “MDR bacteria were defined as bacteria that are non-susceptible to at least one antimicrobial in three or more antimicrobial classes, XDR was defined as non-susceptible to at least one antimicrobial agent in all but two or fewer antimicrobial classes” [17]. Patients were divided into three groups: those with MDR/XDR infections, those with non-MDR/XDR infections, and those who did not develop any bacterial infection in the 3 months after transplant.

Infections were diagnosed based on the current US Centers for Disease Control and Prevention National Healthcare Safety Network criteria [18]. Patients who only showed MDR bacterial colonization (rectal/nasal carriers) were not included among patients with infection. Infections due to Extended-Spectrum Beta-Lactamase (ESBL)-producing Enterobacteriaceae, which did not show resistance against other groups of antibiotics, were included among non-MDR/XDR infections due to the endemic spread of these microorganisms in our clinical setting. The eradication of infection was defined as negative in follow-up cultures coupled with clinical and biochemical parameter improvement.

### 2.3. Analysed Variables

For each patient, we collected general clinical data, hematochemical parameters, treatments received and infection development up to 3 months after heart transplant.

Among general clinical data we considered age, sex, body mass index (BMI), comorbidities, length of hospital and ICU stay, hospitalization in the 90 days prior to heart transplant, previous automatic implantable cardioverter-defibrillator (AICD) implant, previous placement of mechanical circulatory support devices [intra-aortic balloon pump (IABP), left ventricular assist device (L-VAD), extracorporeal membrane oxygenation (ECMO)]. Comorbidities were assessed by means of the Cumulative Illness Rating Scale (CIRS) score [19]. Hematochemical parameters were collected during hospitalization when the transplant was performed, and 1 and 3 months after transplantation: these included white blood cell count, platelet count, C-reactive protein, creatinine, bilirubin, international normalized ratio of prothrombin time, activated partial thromboplastin time, albumin, cyclosporine A/tacrolimus and mycophenolate blood levels. Sequential organ failure assessment score (SOFA score) was calculated for all patients on the days of surgery and of microbiological sample positivity for patients who developed an infection.

Regarding antimicrobial treatment administration, we analyzed antibiotic therapy given in the 7 days previous to heart transplant, in the 48 h after transplant, as well as the antibiotic treatment for each infectious episode. We also recorded immunosuppressive regimens used (and possible replacement of a drug with another immunosuppressant).

Patients were divided according to infection development into 3 subgroups: MDR/XDR infections, non-MDR/XDR infections, and no infection. In-hospital mortality was compared between these 3 groups. Other study outcomes analyzed were eradication of infection, early graft failure, acute transplant rejection, mortality 1 and 3 months after transplantation and length of hospital stay.

### 2.4. Statistical Analysis

Numerical variables were expressed as a median and interquartile range (IQR), while categorical variables were expressed as number and percentage. Categorical variables were compared using Fisher’s exact test or Pearson chi-square, while continuous variables were compared using the Mann-Whitney U-test (two group differences) or Kruskal Wallis (three group differences). Statistical analyses were performed using Graphpad Prism 8 for Microsoft version 8.0.2 (263), using a significance level of 5% and two-tailed tests.

## 3. Results

Some 47 patients who underwent heart transplant (HT) at our center during the study period were included. Baseline features according to infection development are presented in Table 1.

Some 35 patients (74.5%) developed an infection in the 3 months following transplant. Infections due to MDR or XDR bacteria occurred in 29.8% of the entire examined cohort and 40% of transplant recipients who developed an infectious complication. No PDR bacteria were isolated. In 2 patients who had clear signs of infection (clinical characteristics and inflammatory marker elevation), the etiologic cause was not available possibly due to antibiotic treatment prior to performing microbiological tests. We considered these patients among non-MDR/XDR infections since no sign of antimicrobial resistance was evident.

**Figure 1:**
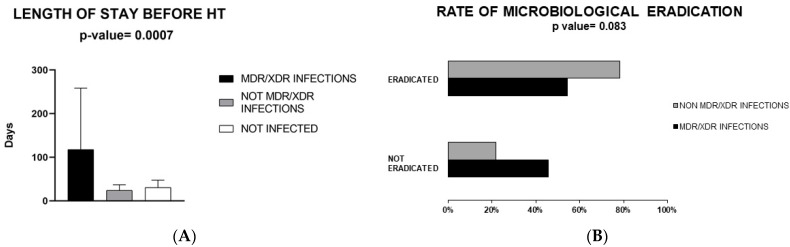
Panel (**A**): Duration of hospitalization before heart transplant based on multidrug-resistant/extensively drug-resistant (MDR/XDR) infection development or no infection. Panel (**B**): Rate of microbiological eradication. Abbreviations: MDR/XDR, multidrug-resistant/extensively drug-resistant; HT, heart transplant.

Thus, a comparison between the following groups was made: (I) patients with MDR/XDR infection [*n* = 14 (29.8%)]; (II) patients with a non-MDR/XDR infection [*n* = 21 (44.7%)]; (III) patients without infection [*n* = 12 (25.5%)]. No significant differences emerged in terms of age, sex and body mass index (Table 1), as well as the nature of the cardiomyopathy that led to the transplantation between these groups. There was no difference in the rate of comorbidities, using CIRS, between the study subgroups. Some 78.6% of MDR patients versus 52.4% of non-MDR patients had a previous hospitalization in the 90 days before surgery (*p* = 0.064). The duration of previous hospitalizations correlated with MDR/XDR infection development (*p* = 0.007) (Figure 1A).

In contrast, ICU stays before HT occurred more often among MDR infection cases but was not significantly associated with a higher risk of developing MDR infection. Also, no differences between the three groups were seen in terms of intracardiac device presence before HT (Table 1).

Most patients received amoxicillin/clavulanic acid as prophylaxis, therefore no correlation was found between the type of antibiotic chosen for surgical prophylaxis or treatment in the previous 7 days of transplantation and the development of MDR/XDR infection (Table 2).

The SOFA score at the time of surgery was higher among MDR patients, although not statistically significant (Supplementary Figure S1). Also, no differences were observed in terms of immune suppressive regimens or their plasma drug levels (Table 1).

As shown in Table 3, the most common infectious syndromes in all patients that developed an infection were lower respiratory tract infections [mostly HAP/VAP (hospital-acquired pneumonia/ventilator-associated pneumonia)] (36.2%), followed by complicated urinary tract infections (cUTI) (24.1%), bloodstream infections (BSIs) (including catheter-related ones) 17.2%, and skin and soft tissue infections (including surgical site infections) (13.8%). Only 6.9% of total infection episodes were mediastinitis. Both BSI and SSTI were significantly more common in MDR/XDR infected patients (*p* = 0.043, *p* = 0.047). In contrast, cUTI were more prevalent in non-MDR/XDR infected patients (*p* = 0.002). Patients with MDR/XDR infection showed higher SOFA scores (calculated on sample positivity day) compared to non-MDR/XDR infection patients (*p* = 0.027). The median time between HT and microbiological sample positivity was not significantly different in MDR/XDR patients (10.5 days vs. 8.5 days in non-MDR/XDR infection patients; *p* = 0.408) (Table 3).

Gram-negative bacteria were the most prevalent etiological agents accounting for 71.4% of total isolates and 62.5% of MDR/XDR isolates. *Klesbsiella pneumoniae* was the most predominant microorganism accounting for 21.4% of total isolates and 20.8% of MDR/XDR isolates. The most common infectious syndrome was HAP/VAP due to *Klebsiella pneumoniae,* accounting for 13.6% of all infectious episodes. Among MDR/XDR infectious episodes, BSI due to methicillin-resistant *Staphylococcus epidermidis* was prevalent, occurring in 16.4% of episodes (Table 4).

The most common resistance mechanism among all *Klebsiella pneumoniae* isolates was the production of KPC-type carbapenemase, expressed by 33.3% of total *Klebsiella pneumoniae* strains (Supplementary Table S1 and data not shown).

Among the gram-negative bacteria in MDR and XDR groups the most common category present was Enterobacteriaceae followed by *Stenotrophomonas maltophilia* which was present in the XDR group. Among Gram positives, *Staphylococcus spp* and *Enterococcus spp* were equally distributed in terms of MDR/XDR and non-MDR/XDR infection and all resistant isolates were MDR.

**Figure 2:**
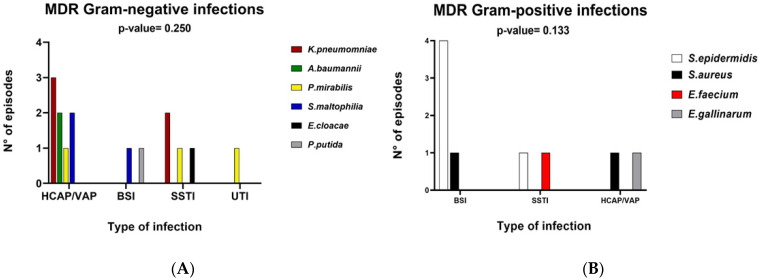
Distribution of MDR/XDR infection based on infectious episodes in bar charts. Panel (**A**) depicts the distribution of gram-negative pathogens and Panel (**B**) depicts distribution of gram-positive pathogens. Abbreviations: MDR, multidrug-resistant; HCAP/VAP, health care-associated/ventilator-associated pneumonia; BSI, bloodstream infection; SSTI, skin and soft tissue infection, UTI, urinary tract infection.

No relationship was found between the type of microorganism and the type of infection in MDR/XDR patients (Figure 2).

In terms of antibiotic treatment, colistin and daptomycin (*p* = 0.039, *p* = 0.017) were more commonly employed in MDR/XDR infections while cefepime in non-MDR/XDR infections (*p* = 0.049). There was a clear trend for a longer duration of treatment in MDR/XDR group compared to non-MDR/XDR infection group (15.5 days for MDR/XDR patients vs. 7.5 days for non-MDR/XDR infection, *p* = 0.057) (Table 3). Also, eradication of infection (defined as surveillance sample negativity in addition to improvement of clinical signs and biochemical parameters) was observed less often in MDR/XDR infection (*p* = 0.083) (excluding the 2 patients who had no proven etiology) (Table 3).

There was no difference between the three groups in terms of laboratory parameters on HT day 1 month and 3 months after transplant, except for total bilirubin levels 1 month after HT which were higher among MDR/XDR patients (*p* = 0.005) (Table 1).

In terms of outcome (Table 5), there were no significant differences in mortality (in-hospital, 1 and 3 months after transplant), early graft failure and acute transplant rejection between the three groups. Interestingly, MDR infection patients had numerically lower rates of acute transplant rejections. Also, MDR infection patients tended to have a better short-term outcome but a worse mid-term prognosis (not significant). However, in the MDR/XDR infection group, the median length of hospital stay was substantially higher (61 days vs. 23 days in non-MDR/XDR infections and 22 days in the no infection group, *p* = 0.005).

## 4. Discussion

Limited published data are available on MDR infections in solid organ transplant recipients [7,15,20,21]. We observed a substantial impact of prior prolonged hospitalization on the development of MDR bacterial infections after HT. Also, a role for a higher rate of previous hospitalizations in patients with MDR infections was evident. More importantly, MDR-infected recipients had significantly longer pre-transplant hospitalization and a higher rate of ICU admission. The influence of greater exposure to the hospital environment on the incidence of MDR infection could plausibly be due to a higher risk of colonization and exposure to antibiotics, as also shown outside of the transplant setting [22,23]. Less important appeared to be the role of medical comorbidities, in contrast to what other studies suggested [24].

It was interesting to observe that SOFA scores on transplantation day and sample positivity day were higher among recipients with MDR/XDR pathogen infections. These data suggest that a state of greater systemic impairment could translate into a greater susceptibility to resistant infections. Consequently, MDR pathogens appear to emerge as the cause of infection mostly in patients with an already poor clinical state.

A further risk factor for infection with MDR bacterial species would be represented by the degree of immune suppression achieved by the recipients [4], once the maintenance therapeutic scheme has been set with the two main associations used (cyclosporine/mycophenolate or tacrolimus/mycophenolate). Although no statistically significant difference was found in the various subgroups, patients with higher plasma levels of immune suppressants had a tendency to develop more frequent MDR/XDR infections.

This hypothesis appears to be further supported by the absence of acute rejection episodes observed in the subgroup of transplant recipients with MDR bacterial infections, at variance with non-MDR and no infection groups, where a few patients did develop acute transplant rejection.

The prevalence of bacterial infections in heart transplant recipients, within 3 months after the procedure, was particularly high, with antibiotic resistance (MDR/XDR) present in 40% of infectious episodes. As in previous studies, Gram-negative bacteria made up the majority of isolates, resulting in pathogenic bacteria in 3 of 4 transplant recipients with infectious complications, as well as presenting a higher rate of multidrug-resistance (62.5% of the MDR/XDR isolates). In particular, carbapenem-resistant *Klebsiella pneumoniae* accounted for 7.1% of total isolates in heart transplant recipients. This is in keeping with a previous publication from our institution showing a continuing risk for MDR/XDR *Klebsiella pneumoniae* invasive infections in our hospital setting [25]. The high prevalence of Gram-negative pathogens in our study suggests that bacterial translocation from the gut could be a mechanism of bloodstream invasion, as also shown in liver transplant recipients [26]. Likewise, these BSI were not associated with a worse prognosis [26].

A higher proportion of SSTI and BSI episodes were due to MDR pathogens, explaining the need to focus on preventing surgical site infections and intravascular catheter colonization, both potential sources of serious infections. The risk of developing a surgical site infection following heart surgery may be up to 7.9% [27,28], but in heart transplant recipients this risk seems to be higher, as is also shown by our results. In order to prevent catheter-related BSI, the use of chlorhexidine bathing, sterile handling of lines and frequent substitution of intravascular catheters are of particular importance.

Patients with MDR infections had a longer duration of antimicrobial therapy and of hospitalization suggesting a greater difficulty in infection eradication. However, other important outcomes were not significantly affected by MDR infections, including mortality, although a longer hospitalization increases health-care costs [29]. Although not significant, mortality was higher in both non-MDR/XDR and MDR/XDR infection patients compared to non-infected patients, in line with other studies observing an infection/related mortality following heart transplant ranging from 18% to 36% [6,30]. Therefore, preventing the development of MDR/XDR infection in transplant recipients is of great importance. As patients who undergo frequent hospitalization are at risk of developing future MDR infection, it is important to consider this feature of clinical history in the pre-transplant evaluation. These patients, especially those who are already colonized with MDR pathogens, should not undergo an over-immunosuppression and possibly receive modulated doses of calcineurin inhibitors as a preventive measure which, in addition to infection control interventions, may play an important role in lowering the rate of developing MDR infections [31]. The results of this study allowed us to tailor the perioperative antimicrobial prophylaxis protocol based on the most important observed risk factors for MDR/XDR infections. This study has several limitations. It was a retrospective case/control study including a relatively low number of patients. Additionally, data on frequency and timing of any previously treated infection or previous cardiac surgery were not available. Due to the relatively low number of patients included, we could not dissect the drivers of a specific pattern of resistance (MDR vs. XDR), which is a further limitation of our study. Finally, as this study was conducted at a single institution, the results may not be applicable to other settings with different local epidemiology.

## 5. Conclusions

MDR/XDR infections tend to affect more seriously ill HT recipients with a history of prolonged hospitalization, causing a further significant increase of hospitalization length and a low rate of eradication. Developing an MDR/XDR infection triggers a vicious circle: the longer the hospitalization the higher the risk of developing MDR/XDR infection. On the other hand, developing MDR/XDR infection puts the patient at a higher risk for a longer hospitalization. Since MDR infections frequently present as BSI and surgical site infections in HT patients, we emphasize the need for the prevention of contamination of central venous catheters and surgical sites, both common sources of infections due to these difficult-to-treat pathogens.

## Figures and Tables

**Table 1:** Patients’ baseline characteristics and laboratory parameters within 3 months after heart transplant.

Variables	MDR/XDR Infections	Non-MDR/XDR Infections	Not Infected	*p*
**Patients**	14 (29.8)	21 (44.7)	12 (25.5)	
**Recipient age, years**	47 (4–64)	53 (11–68)	45 (18–63)	0.168
**Recipient gender**				0.313
Male	13 (92.9)	15 (71.4)	9 (75)	
Female	1 (7.1)	6 (28.6)	3 (25)	
**Body mass index, kg/m^2^**	22.8 (16.6–33.3)	24.7 (17.6–34.9)	26 (18.3–34.3)	0.196
**Comorbidities and CIRS**				
Obesity	1 (7.1)	3 (14.3)	4 (33.3)	0.248
Chronic kidney disease	7 (50)	6 (28.6)	3 (25)	0.39
Diabetes	3 (21.4)	5 (23.8)	2 (16.7)	>0.999
Chronic pulmonary disease	3 (21.4)	8 (38.1)	3 (25)	0.656
Chronic liver disease	1 (7.1)	3 (14.3)	0	0.457
Arterial hypertension	7 (50)	15 (71.4)	7 (58.3)	0.375
Dyslipidemia	8 (57.1)	11 (52.4)	8 (66.7)	0.7
Solid tumors	2 (14.3)	2 (9.5)	2 (16.7)	0.864
Cerebrovascular accident/transient ischemic attack	2 (14.3)	5 (23.8)	1 (8.3)	0.616
Cumulative Illness Rating Scale (CIRS)				
	12.5 (5–22)	15 (5–21)	12 (4–17)	0.099
**Previous hospitalization < 90 days**	11 (78.6)	11 (52.4)	4 (33.3)	0.064
**Recipient location right before HT**				0.699
Home	8 (57.1)	14 (66.7)	7 (58.3)	
Medical/surgical units	3 (21.4)	3 (14.3)	4 (33.3)	
Intensive care units	3 (21.4)	4 (19)	1 (8.3)	
**Intensive Care Unit stay before HT**				0.442
Yes	6 (42.9)	5 (23.8)	3 (25)	
No	8 (57.1)	16 (76.2)	9 (75)	
**AICD/MCS-devices implanted before HT**				
AICD before HT				
VAD before HT	11 (78.6)	19 (90.5)	41 (87.23)	0.674
ECMO before HT	3 (21.4)	1 (4.8)	6 (12.77)	0.218
IABP before HT	1 (7.1)	3 (14.3)	4 (8.51)	0.457
Any MCS-device	2 (14.3)	1 (4.8)	4 (8.51)	0.802
	4 (28.6)	4 (19)	11 (23.4)	0.655
**IMMUNOSUPPRESSIVE REGIMENS**				
Prednisone				
Cyclosporine A + mycophenolate	13 (92.9)	16 (76.2)	11 (91.7)	0.376
Tacrolimus + mycophenolate	8 (57.1)	11 (52.4)	7 (58.3)	0.919
Cyclosporine A	3 (21.4)	6 (28.6)	3 (25)	0.892
Cyclosporine A → Tacrolimus	3 (21.4)	4 (19)	1 (8.3)	0.639
Tacrolimus → Cyclosporine A	0	1 (4.8)	0	1
	0	0	1 (8.3)	0.255
**Immunosuppressant blood level**				
Cyclosporine A, ng/dL	264.5 (121–328.5)	219.5 (98–493)	220.8 (115–308.5)	0.342
Tacrolimus, ng/mL	8.6 (7.2–9.7)	7.8 (6.2–12.4)	6.4 (1.4–8.1)	0.348
Mycophenolate, μg/mL	1.1 (0.8–2)	1.3 (0.4–2.9)	1.2 (0.8–2)	0.552
**Laboratory data**				
Glomerular Filtration Rate (CKD-EPI) pre-HT, mL/min/1.83 m^2^	61.5 (19–156)	78 (26–128)	90 (49–158)	0.388
Creatinine pre-HT, mg/dL				
Creatinine onset 1st infection, mg/dL	1.4 (0.5–3.5)	1.1 (0.6–2.5)	0.9(0.5–1.6)	0.489
Creatinine EOH, mg/dL	1.1 (0.5–2.3)	1 (0.4–2.3)	1.1 (0.6–2.9)	0.4
Creatinine 1 m post-HT, mg/dL	0.9 (0.2–2.6)	0.9 (0.2–2)		0.904
Creatinine 3 m post-HT, mg/dL	1.1 (0.5–3.7)	0.9 (0.3–2.2)	1.1 (0.8–1.6)	0.573
Bilirubin pre-HT, mg/dL	1.2 (0.5–1.9)	1 (0.5–1.5)	1 (0.6–1.3)	0.72
Bilirubin onset 1st infection, mg/dL	1.3 (0.6–3.3)	1 (0.2–2.7)	1 (0.4–3.6)	0.233
Bilirubin EOH, mg/dL	3.45 (0.7–35.3)	1.9 (0.4–5)	1.1 (0.4–6)	0.096
Bilirubin 1 m post-HT, mg/dL	1.7 (0.7–35)	1.9 (0.5–21.8)		0.255
Bilirubin 3 m post-HT, mg/dL	1.9 (0.8–2.7)	1 (0.4–6.1)	0.7 (0.4–2.1)	**0.005**
INR pre-HT	0.9 (0.4–2.1)	0.6 (0.3–1.4)	0.7 (0.4–1.2)	0.264
INR onset 1st infection	2.31 (1.05–6.51)	1.76 (0.95–4.27)	1.38 (1.02–3.4)	0.206
INR EOH	1.08 (0.97–1.99)	1.21 (1.02–3.91)	1.01 (0.97–2.94)	0.781
INR 1 m post-HT	1.13 (0.95–1.54)	1.11 (0.99–5.25)		0.765
INR 3 m post-HT	1.16 (0.94–1.36)	1.05 (0.99–3.21)	1.04 (0.94–1.23)	0.282
Lymphocytes pre-HT, u/mmc	1.02 (0.95–1.57)	1.03 (0.92–2.44)	1.02 (0.92–1.08)	0.433
Lymphocytes post-HT, u/mmc	1.31 (0.81–2.25)	1.56 (0.77–3.21)	1.88 (0.8–4.83)	0.262
Lymphocytes 1 m post-HT, u/mmc	0.49 (0.08–2.72)	0.69 (0.09–3.21)	0.32 (0.11–1.53)	0.588
WBC > 15.000/mmc during hospital stay	0.54 (0.12–2.53)	0.67 (0.2–1.8)	0.77 (0.49–1.96)	0.561
PLT < 10.000/mmc during hospital stay	8 (57.1)	17 (81)	10 (83.3)	0.205
	2 (14.3)	1 (4.8)	0	0.445

Data are expressed as median and interquartile range, or number and percentage. Abbreviations: AICD, automatic implantable cardioverter-defibrillator, ECMO, Extracorporeal membrane oxygenation; EOH, end of hospitalization; IABP, intra-aortic balloon pump; HT, heart transplant; INR, international normalized ratio; M, month; MCS, Mechanical Circulatory Support; MDR/XDR, multidrug-resistant/extensively drug-resistant; PLT, Platelets; VAD, ventricular assist device; WBC, white blood cells. The number reported for AICD/MCS devices implanted before HT refers to the number of patients.

**Table 2:** Antibiotic treatment before heart transplant and as surgery prophylaxis.

	mdr/xdr Infections (*n* = 14)	Non-mdr/xdr Infections (*n* = 21)	Not Infected (*n* = 12)	*p*
**Ab therapy previous 7 days; *n* (%)**	**4 (28.6)**	**4 (19)**	**4 (33.3)**	ns
Amoxicillin/Clavulanic acid	1 (7.1)	2 (9.5)	2 (16.7)	0.704
Meropenem	1 (7.1)	1 (4.8)	0	0.999
Linezolid	0	1 (4.8)	1 (8.3)	0.728
Vancomycin	0	1 (4.8)	0	1
Cefepime	0	1 (4.8)	0	1
Piperacillin/tazobactam	0	1 (4.8)	0	1
Levofloxacin	0	1 (4.8)	0	1
Colistin	1 (7.1)	0	0	0.553
Daptomycin	1 (7.1)	0	2 (16.7)	0.093
Minocycline	1 (7.1)	0	0	0.553
Trimethoprim/sulfamethoxazole	1 (7.1)	0	1 (8.3)	0.300
Gentamicin	0	0	1 (8.3)	0.255
**Antibiotic prophylaxis;*n* (%)**				
Amoxicillin/Clavulanic acid	12 (85.7)	16 (76.2)	11 (91.7)	0.616
Colistin	0	0	1 (8.3)	0.255
Meropenem	1 (7.1)	1 (4.8)	0	0.999
Vancomycin	2 (14.3)	4 (19)	1 (8.3)	0.872
Cefepime	0	1 (4.8)	0	1
Daptomycin	0	1 (4.8)	0	1

Abbreviations: ns, non-significant.

**Table 3:** Infection characteristics and treatment in heart transplanted patients.

Parameters	All	mdr/xdr Infections	Non-mdr/xdr Infections	*p*
Patients with any infectious episodes *	**35 (74.5)**	**14 (40)**	**21 (60)**	0.135
**Total infectious episodes, n**	58	24	34	
**Types of infectious episodes**				
Pneumonia	21 (36.2)	10 (41.7)	11 (32.3)	0.577
Bloodstream infection (BSI)	10 (17.2)	7 (29.2)	3 (8.8)	**0.043**
Skin and soft tissue infection (SSTI)	8 (13.8)	6 (25)	2 (5.9)	**0.047**
Complicated Urinary tract infection (cUTI)	14 (24.1)	1 (1.8)	13 (38.2)	**0.002**
Mediastinitis	4 (6.9)	0	4 (11.8)	0.072
Unknown	1(1.7)	0	1 (3)	ns
SOFA **score at the time of 1st infection onset**	7 (1–15)	8 (4–13)	4.5 (1–15)	**0.027**
**days between ht and 1st isolation**	9.5 (0–54)	10.5 (3–44)	8.5 (0–54)	0.408
**eradication of infectious episodes**	38 (65.5)	13 (54.2)	25 (78.1) **	0.083
**antibiotic therapy**				
Amikacin	1 (1.7)	1 (4.1)	0	ns
Amoxicillin/clavulanic acid	6 (10.3)	1 (4.1)	5 (14.7)	0.366
Aztreonam	1 (1.7)	1 (4.1)	0	ns
Cefazolin	1 (1.7)	0	1 (2.9)	ns
Cefepime	5 (8.6)	0	5 (14.7)	**0.049**
Cefixime	1 (1.7)	0	1 (2.9)	ns
Ceftazidime/avibactam	2 (3.4)	2 (8,2)	0	0.153
Ciprofloxacin	2 (3.4)	0	2 (5.8)	ns
Colistin aerosol	4 (6.8)	3 (12.3)	1 (2.9)	ns
Colistin iv	8 (13.7)	6 (25)	2 (2.9)	**0.039**
Cotrimoxazole	8 (13.7)	4 (16.4)	4 (11.7)	ns
Daptomycin	6 (5.1)	5 (20.8)	1 (2.9)	**0.017**
Ertapenem	1 (1.7)	1 (4.1)	0	ns
Gentamicin ev	3 (5.1)	3 (12.3)	0	ns
Levofloxacin	4 (6.8)	0	4 (11.7)	0.082
Meropenem	9 (15.5)	4 (16.4)	5 (14.7)	ns
Piperacillin/tazobactam	8 (13.7)	3 (12.3)	5 (14.7)	ns
Teicoplanin	5 (8.6)	0	5 (14.7)	0.049
Tigecycline	2 (3.4)	1 (4.1)	1 (2.9)	ns
Duration of therapy, days	10 (2–61)	15.5 (3–61)	7.5 (2–38)	**0.057**

Data are expressed as median and interquartile range, or number and percentages; Abbreviations: iv, intravenous; MDR/XDR, multidrug-resistant/extensively drug-resistant; SOFA, Sequential Organ Failure Assessment; ns, non-significant. * Percentages in rows. ** Two patients without microbiological etiology excluded.

**Table 4:** Etiology of infections in heart transplanted patients.

Isolated Pathogens	All (*n* = 56)	MDR/XDR (*n* = 24)	Non-MDR/XDR (*n* = 32)
**HAP/VAP**			
**Gram-negative**			
*Klebsiella pneumoniae*	8 (13.6)	3 (12.3)	5 (15.6)
*Acinetobacter baumannii*	2 (3.4)	2 (8.2)	
*Escherichia coli*	2 (3.4)		2 (6.2)
*Serratia marcescens*	1 (1.7)		1 (3.1)
*Haemophilus influenzae*	1 (1.7)		1 (3.1)
*Stenotrophomonas maltophilia*	1 (1.7)	1 (4.1)	
**Gram-positive**			
*Staphylococcus aureus*	3 (5.3)	1 (4.1)	2 (6.2)
*Enterococcus gallinarum*	1 (1.7)	1 (4.1)	
*Enterococcus faecium*	1 (1.7)	1 (4.1)	
**cUTI**			
**Gram-positive**	-	-	-
**Gram-negative**			
*Escherichia coli*	5 (8.9)		5 (15.6)
*Proteus mirabilis*	3 (5.3)	1 (4.1)	2 (6.2)
*Morganella morgani*	2 (3.4)		2 (6.2)
*Pseudomonas aeruginosa*	1 (1.7)		1 (3.1)
*Klebsiella pneumoniae*	1 (1.7)		1 (3.1)
*Citrobacter koseri*	1 (1.7)		1 (3.1)
*Enterobacter cloacae*	1 (1.7)		1 (3.1)
**BSI**			
**Gram-positive**			
*Staphylococcus epidermidis*	4 (6.8)	4 (16.4)	
*Staphylococcus lugdunensis*	1 (1.7)		1 (3.1)
*Staphylococcus aureus*	1 (1.7)	1 (4.1)	
*Enterococcus faecium*	1 (1.7)		1 (3.1)
**Gram-negative**			
*Stenotrophomonas maltophilia*	2 (3.4)	2 (8.2)	
*Klebsiella pneumoniae*	2 (3.4)	1 (4.1)	1 (3.1)
*Pseudomonas putida*	1 (1.7)	1 (4.1)	
*Serratia marcescens*	1 (1.7)		1 (3.1)
*Proteus mirabilis*	1 (1.7)	1 (4.1)	
**SSTI**			
**Gram-positive**			
*Enterococcus faecalis*	2 (3.4)		2 (6.2)
*Staphylococcus epidermidis*	1 (1.7)	1 (4.1)	
*Staphylococcus aureus*	1 (1.7)		1 (3.1)
**Gram-negative**			
*Klebsiella pneumoniae*	1(1.7)	1 (4.1)	
*Enterobacter cloacae*	1 (1.7)	1 (4.1)	
*Proteus mirabilis*	1 (1.7)	1 (4.1)	
*Escherichia coli*	1 (1.7)		1 (1.7)
**Total Isolates**			
**Gram-positives**	16 (28.5)	9 (37.5)	7 (21.8)
*Staphylococcus epidermidis*	5 (8.9)	5 (20.8)	
**Gram-negatives**	40 (71.4)	15 (62.5)	25 (78.1)
*Klebsiella pneumoniae*	12 (21.4)	5 (20.8)	7 (21.8)

Data are expressed as numbers and percentages. There were no statistically significant differences between the 2 groups (MDR/XDR vs. non-MDR/XDR). Abbreviations: MDR/XDR, multidrug-resistant/extensively drug-resistant; HAP/VAP, hospital-acquired pneumonia/ventilation-associated pneumonia; BSI, bloodstream infection; cUTI, complicated urinary tract infection; SSTI, skin and soft tissue infection.

**Table 5:** Outcome of heart transplant patients according to infection features.

Endpoints	Mdr/xdr Infections (*n* = 14)	Non-mdr/xdr Infections (*n* = 21)	Not Infected (*n* = 12)	*p*
**Early Graft Failure**	3 (21.4)	7 (33.3)	2 (16.7)	0.524
**Add Device post-HT**	3 (21.4)	5 (23.8)	2 (16.7)	0.890
**Acute Transplant Rejection**	0	5 (23.8)	2 (16.7)	0.150
**30-day mortality**	2 (14.3)	6 (28.6)	2 (16.7)	0.541
**In-hospital mortality**	4 (28.6)	7 (33.3)	2 (16.7)	0.586
**3-month mortality**	6 (42.9)	7 (33.3)	2 (16.7)	0.354
**Length of hospitalization, days**	61 (22–431)	23 (9–90)	22 (6–79)	**0.005**

Data are expressed as median and interquartile range, or number and percentages. Abbreviations: MDR/XDR, multidrug-resistance/extensively drug-resistance.

## Data Availability

The dataset used for this study is available on request to the corresponding Author.

## Supplementary Materials

The following are available online at https://www.mdpi.com/article/10.3390/microorganisms9061210/s1, Figure S1: Scatter plot showing the relationship between the sequential organ failure assessment (SOFA) score values at surgery day among the three study groups and at the onset of the first infectious episode in patients with infection. Table S1. Phenotypic characteristics of MDR/XDR pathogens from heart transplanted patients.
