# Supplementary figures and images for: Risk Factors and Outcome of Multidrug-Resistant Infections after Heart Transplant: A Contemporary Single Center Experience
Source: Microorganisms. 2021 Jun 3;9(6):1210. doi: 10.3390/microorganisms9061210 (PMC8230299; doi:10.3390/microorganisms9061210)

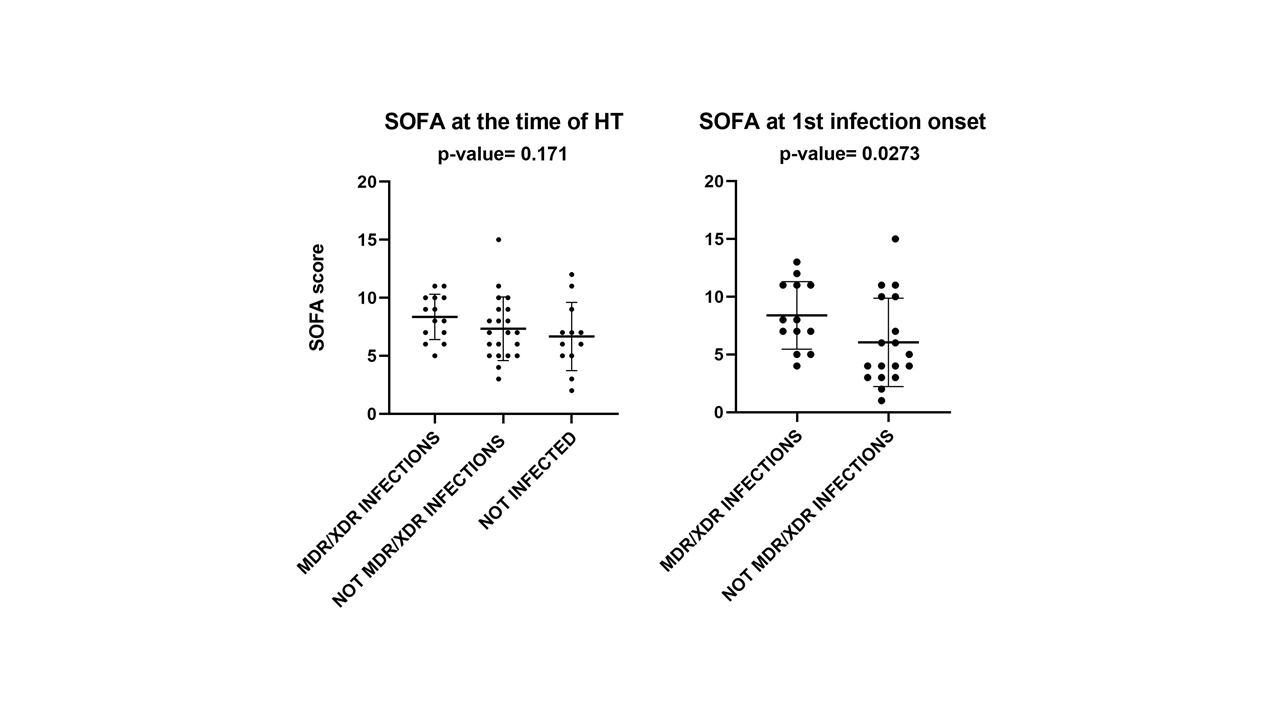

Supplement: Supplementary file 1 [file microorganisms-09-01210-s001.zip › Supplementary Figure 1.tif]
